# Supplementary material for: Multimodality Imaging of Moyamoya Disease: A Practical Guide for Neuroradiologists Based on a Case Report
Source: Reports (MDPI). 2025 Nov 11;8(4):232. doi: 10.3390/reports8040232 (PMC12643450; doi:10.3390/reports8040232)
Supplement: Supplementary file 1 [file reports-08-00232-s001.zip › reports-3865872-supplementary.pdf]

Table S1: CT imaging protocol of the brain.

**CT Protocol**	**NECT**	**CTA**	**VR 3D**
Acquisition modality	helical	helical	VR reconstruction
Slices thickness (mm)	1.25	0.625	0.625
FOV (cm)	23	23	14
kV	120	100	-
mA	170-330	200-330	-
Pitch	0.531:1	0.992:1	-
Coverage speed (mm/s)	13.28	158.75	-
Reformat	-	-	small vessels analysis
Time scan (s)	13.6	3.1	-
DLP (mGy*cm)	715.68	291.64	-

NECT = non enhanced computed tomography, CTA = computed tomography angiography, VR = volume rendering

Table S2: technical parameters of MR imaging protocol of the brain.

MRI Protocol	T2W FSE	3D FLAIR	2D FLAIR	T1W SE	T2*W GRE	DWI SE EPI	TOF
Acquisition plane	axial	sagittal	axial	axial	axial	axial	axial
Repetition time/Echo time (ms)	7302/105	5000/120	9412/120	540/11	879/13.5	7837/80	29 x 6.8
Echo train length	21	159	26	-	-	-	
NEX	2	1	0.5	2	1	2	0.84
Slice thickness (mm)	4	1.3	4	4	4	4	0.8
Interslice gap (mm)	0.5	-	0.5	1	0.5	0.5	-
Field of view (mm)	353 x 250	375 x 266	353 x 250	353 x 250	353 x 250	353 x 250	310 x 220
Matrix	384 x 320	200 x 200	320 x 224	288 x 192	288 x 200	96 x 128	332 x 244
Frequency direction	Anterior to posterior	Superior to inferior	Anterior to posterior	Anterior to posterior	Anterior to posterior	Right to left	Anterior to posterior
b-value (s/mm²)	-	-	-	-	-	0–1000	-
Scan time	2 min 34 s	5 min 28 s	2 min 40 s	3 min 43 s	2 min 32 s	1 min 34 s	4 min 50 s

Table S2: technical parameters of MR imaging protocol of the brain.

T1W = T1-weighted, T2W = T2-weighted, FSE = fast spin-echo, FLAIR = fluid attenuated inversion recovery, GRE = gradient echo, DWI = diffusion-weighted imaging, SE = spin-echo, EPI = echoplanar imaging, TOF = time of flight
